# Prospective multicenter study of the epidemiological features of emergency patients with overdose of over‐the‐counter drugs in Japan

**DOI:** 10.1002/pcn5.225

**Published:** 2024-07-15

**Authors:** Ryoko Kyan, Yoshito Kamijo, Saeko Kohara, Michiko Takai, Takuya Shimane, Toshihiko Matsumoto, Hidetada Fukushima, Shogo Narumi, Takuyo Chiba, Toshiki Sera, Norio Otani, Yasumasa Iwasaki

**Affiliations:** ^1^ Department of Clinical Toxicology Saitama Medical University Hospital Iruma‐gun Saitama Japan; ^2^ Department of Critical Care Medicine and Trauma National Hospital Organization Disaster Medical Center Tachikawa Tokyo Japan; ^3^ Department of Drug Dependence Research National Institute of Mental Health, National Center of Neurology and Psychiatry Kodaira Tokyo Japan; ^4^ Department of Emergency and Critical Care Medicine Nara Medical University Kashihara Nara Japan; ^5^ Emergency department Saga University Hospital Saga Saga Japan; ^6^ Department of Emergency Medicine International University of Health and Welfare Narita Hospital Narita Chiba Japan; ^7^ Department of Emergency and Critical Care Medicine Hiroshima Prefectural Hospital Hiroshima Hiroshima Japan; ^8^ Department of Emergency and Critical Care Medicine St. Luke's International Hospital Chuo‐ku Tokyo Japan; ^9^ Department of Emergency Medical National Hospital Organization Kure Medical Center Aoyama Hiroshima Japan

**Keywords:** abuse, dependence, excessive use, overdose, over‐the‐counter drugs

## Abstract

**Aim:**

To investigate the epidemiological characteristics of patients presenting to the emergency department with an overdose of over‐the‐counter (OTC) drugs.

**Methods:**

A questionnaire survey was conducted to examine the sociodemographic characteristics of patients with OTC drugs overdoses visiting emergency departments at eight sites across the country. The patients were divided into “habitual” and “nonhabitual” groups according to their history of OTC drugs overdose. Student's *t*‐test or Welch's *t*‐test was performed for numerical variables, and Pearson's *χ* ^2^ test was performed for dichotomous and nominal variables between the two groups.

**Results:**

Of the 124 patients included in this study, 79% were women. The habitual (26.6%) and the nonhabitual (73.4%) groups showed no differences in sex, occupation, cohabitants, history of mental illness, or history of alcohol consumption or smoking; however, those in the habitual group were significantly younger. The proportion of OTC drugs obtained from physical stores was higher in the habitual group, whereas the nonhabitual group used more household medicines. Suicide and self‐harm were more common reasons for overdose in the nonhabitual group. Antipyretic analgesics were significantly more common in the nonhabitual group, whereas antitussive expectorants and antihistamines were significantly more common in the habitual group.

**Conclusion:**

This is the first multicenter study to determine the status of OTC drugs overdose patients treated at emergency departments of medical facilities in Japan. To prevent new overdoses of OTC drugs, continued detailed epidemiologic studies of patient backgrounds and drug acquisition routes, and investigation of the components of OTC drugs that cause dependency are necessary.

## INTRODUCTION

The World Health Organization (WHO) has recommended self‐medication and self‐care since 1998.^1^ Consequently, over‐the‐counter (OTC) drugs can be purchased without a prescription from a physician. The Japanese government has emphasized the importance of self‐medication in response to rising medical costs and lifestyle‐related diseases. Previously, OTC drugs could only be obtained from pharmacists in pharmacies and drugstores, but the enforcement of the revised Pharmaceutical Affairs Law in June 2014 lifted the ban on their online sales. Between April 2020 and March 2022, when a state of emergency was declared because of the coronavirus pandemic and strict measures were implemented to prevent its spread, hospital visits for common illnesses and obtaining a prescription for medication became difficult. As a result, the demand for OTC drugs, especially cold remedies, antitussives, and analgesics, increased, and OTC drugs have become increasingly indispensable in daily life. Subsequently, owing to their easy accessibility, OTC drugs abuse has emerged as a social problem,^2^, ^3^ especially among the younger population. Notably, OTC drugs abuse has increased significantly in recent years.

Although multiple studies of OTC drugs dependence and abuse have been conducted, especially in the field of psychiatry,^3^, ^4^ except for single‐center reports, there are no large epidemiologic studies based in emergency departments. Some OTC drugs overdose patients have no history of psychiatric consultation. In a single‐center study by Hirose et al.,^5^ 58% of OTC drugs overdose patients presenting to emergency departments had no history of psychiatric consultation, and 30% were younger than 20 years of age. Since there is a possibility that adolescents may not receive appropriate mental health care even when they are in distress, it is extremely important to conduct surveys that include cases that do not involve psychiatric institutions to promote measures against dependence and abuse of OTC drugs, which will become a social problem in the future. We therefore conducted a large‐scale epidemiologic study based in the emergency department to clarify the background of OTC drugs overdose. In addition, the composition of OTC drugs in Japan differs from that of other countries. For instance, the prevalence of multidrug formulations, which is uncommon in other countries, the inclusion of ingredients, such as ephedrine, codeine, dihydrocodeine, bromovaleryl urea, pseudoephedrine, methylephedrine, which are listed by the Ministry of Health, Labor and Welfare with abuse potential, in OTC drugs and their ready availability to the general public, and the inclusion of caffeine as an ingredient in OTC drugs are all problems unique to Japan. From this perspective, Japan's own epidemiological studies would be highly beneficial.

## METHODS

### Subjects

The Japanese Society of Clinical and Analytical Toxicology is an academic society of clinical and analytical toxicologists, consisting of physicians, pharmacists, and other researchers affiliated with hospitals, universities, and institutions. On February 16, 2021, email letters were sent to 16 emergency medical facilities with emergency physicians who were members of the society, requesting their participation in an epidemiological study on overdose of OTC drugs on a large scale. The study participants were patients who presented to an emergency facility between May 2021 and December 2022 due to an overdose of OTC drugs and who agreed to participate in the study, even if the patient was underage. In this study, we obtained written consent from all patients. In cases involving underage patients, we provided explanations using assent statements and obtained written consent from both the patients and their guardians. OTC drugs are medications that can be purchased at pharmacies without a prescription from a doctor. Although caffeine tablets can be purchased in Japan as either OTC drugs or supplements, both types of caffeine were included in this study. Overdose or excessive use was defined as taking a dose higher than the recommended dosage. Patients who confessed overdosing with OTC drugs, had witnessed overdosing of these drugs, or were found to be in possession of residual drugs at the scene were included in this study. In addition, as the aim of this study was to examine the real situation of OTC drug overdoses, we did not exclude patients who co‐ingested prescription drugs and/or alcohol with OTC drugs. The doses of OTC drugs were estimated from patients' confessions, empty boxes, bottles, or packages found around the patient at the scene.

### Measures

Participating emergency facilities were emailed a questionnaire that included close‐ended questions regarding the following: patient's age, sex, occupation, housemates, history of mental health problems, social history of alcohol and smoking, history of habitual excessive use of OTC drugs, route of access to drugs/information, commercial names and consumed amounts of OTC drugs, concomitantly consumed drugs/substances, and the purpose of this overdose. A pilot questionnaire was tested for simplicity in terms of understanding and the time required for completion. Physicians at the participating emergency facilities were required to search for all patients fulfilling the above conditions and to complete the questionnaire for every appropriate patient. All questionnaires were collected and analyzed at the Department of Clinical Toxicology, Saitama Medical University Hospital. This study was approved by the Institutional Review Board of the Ethics Committee of the Saitama Medical University Hospital (2021‐064).

### Data analyses

The sociodemographic characteristics of patients with OTC drugs overdose were evaluated. Patients were divided into two groups, the “habitual group,” which included patients with a history of habitual excessive use of OTC drugs and the “nonhabitual group,” which included patients without a history of habitual excessive use. In the survey questionnaire, “history of habitual excessive use of OTC drugs” was defined as a case in which the patient had three or more overdoses and routinely overdosed for other than the intended purpose. Student's *t*‐test or Welch's *t*‐test was performed for numerical variables, and Pearson's *χ* ^2^ test was performed for dichotomous and nominal variables between the two groups using IBM® SPSS® version 29.0 with an alpha level of 0.05 as statistically significant.

## RESULTS

### Study population

A total of 124 patients from eight emergency medical facilities who consented to participate in this study (50.0%) were included. We did not exclude any patients. Of the total patients, 26 (21%) were men and 98 (79%) were women, with a median age of 22.0 (12–85) years. In terms of occupation, 43 (34.7%) were students, 32 (25.8%) were full‐time workers, 20 (16.1%) were part‐time workers, 11 (8.9%) were unemployed, five (4.0%) were stay‐at‐home spouses, three (2.4%) were on leave or leave of absence from school, one (0.8%) was searching for a job, one (0.8%) was freelance, and eight (6.5%) were others. A total of 102 (82.3%) patients were unmarried, 12 (9.7%) were married, four (3.2%) were divorced, two (1.6%) were in a common‐law relationship, and three (3.2%) had unknown status. Furthermore, 87 (70.2%) patients lived with family members, 12 (9.7%) with a partner, two (1.6%) with friends, two (1.6%) with others, 18 (14.5%) lived alone, and three (2.4%) had unknown status.

### Comparison between the habitual and nonhabitual groups

Notably, 33 (26.6%) patients with a history of habitual excessive use of OTC drugs were included in the habitual group, whereas 91 (73.4%) patients without a history of habitual excessive use were included in the nonhabitual group. We compared the patients' backgrounds between the two groups (Tables 1 and 2). Although no significant differences were observed in terms of sex, occupation, housemates, history of mental health problems, and history of drinking or smoking, the patients in the habitual group were significantly younger (*t* (122) = 2.11, P = .037). Moreover, those in the habitual group obtained more information about OTC drugs from the internet (*x* ^2^ (1) = 6.14, P = .013) or friends (*x* ^2^ (1) = 17.39, P < .001). In addition, more patients in the habitual group obtained OTC drugs from physical stores (such as pharmacies and drugstores) (*x* ^2^ (1) = 6.74, P = .009). In contrast, those in the nonhabitual group tended to use household medicines (*x* ^2^ (1) = 3.78, P = .052). Suicide and self‐harm were the common reasons for overdose in the nonhabitual group (*x* ^2^ (1) = 9.21, P = .002), whereas purposes other than suicide or self‐harm were significantly more common in the habitual group (*x* ^2^ (1) = 32.27, P < .001). Purposes other than suicide or self‐harm included escapism, relief from mental distress, relaxation, and no particular reason (Table 1).

**Table 1:** Comparison of the backgrounds of the habitual and nonhabitual groups.

	Nonhabitual, *n* = 91	Habitual, *n* = 33	*P*‐value
Age (mean)	27.02	21.82	0.037
Sex (female)	70	28	0.338
Occupation (yes)	76	27	0.824
Housemate (yes)	74	29	0.602
Mental problems (yes)	50	21	0.387
Social history			
Alcohol (yes)	50	12	0.083
Smoking (yes)	18	2	0.088
Concomitant drugs/substances (yes)	35	12	0.732
Route of access to drugs (total 129)			
Physical store (yes)	58	29	0.009
Family owned (yes)	26	2	0.008
Internet purchase (yes)	8	4	0.579
Other (yes)	1	1	0.451
Route of access to information (total 131)			
Internet			
Physical store (yes)	30	19	0.013
Social networking system (yes)	22	3	0.064
Friends (yes)	13	9	0.094
Other (yes)	0	6	<0.001
Purpose of use (total 191) (yes)	24	5	0.192
Suicide/self‐injury (yes)	78	20	0.002
Other (yes)	15	23	<0.001
Escapism (yes)	5	6	
Relief of mental distress (yes)	7	7	
Relaxation (yes)	2	13	
Nothing in particular (yes)	2	3	

Among the categories of OTC drugs, antipyretic analgesics were significantly more common in the nonhabitual group (*x* ^2^ (1) = 4.63, P = 0.031), whereas antitussive expectorants (*x* ^2^ (1) = 12.08, P ≤ 0.001) and antihistamines (*x* ^2^ (1) = 9.07, P = 0.003) were significantly more common in the habitual group. As an ingredient in OTC drugs, dextromethorphan was significantly more common (*x* ^2^ (1) = 10.47, P = 0.001) in the habitual group (Table 2).

**Table 2:** Comparison of the drugs taken by the habitual and the nonhabitual groups.

	Nonhabitual, *n* = 91	Habitual, *n* = 33	*P*‐value
Time from intake to hospital (mean)	344.42	457.83	0.135
Total number of tablets taken (mean)	101.22	121.77	0.392
Total number of drug category (mean)	1.58	1.39	0.267
Drug category (total 144)			
Antipyretic analgesics (yes)	32	5	0.031
Cold medicine (yes)	18	8	0.590
Antitussives and expectorants (yes)	16	16	<0.001
Hypnotic sedative (yes)	14	2	0.171
Antivertigo medicines (yes)	6	1	0.447
Antihistamine (yes)	5	8	0.003
Agents to prevent drowsiness (caffeine preparation) (yes)	9	0	0.061
Herbal medicine (yes)	4	0	0.221
Contains ingredients (total 386)			
Anhydrous caffeine (yes)	65	21	0.406
DL‐methyl ephedrine hydrochloride (yes)	37	19	0.094
Chlorpheniramine maleate (yes)	33	15	0.353
Dihydrocodeine phosphate/codeine phosphate hydrate (yes)	32	15	0.297
Diphenhydramine hydrochloride/diphenhydramine salicylate (yes)	24	11	0.447
Acetaminophen (yes)	22	10	0.491
Ibuprofen (yes)	26	4	0.059
Dextromethorphan hydrobromide hydrate (yes)	7	10	0.001
Aspirin (acetylsalicylic acid) (yes)	11	2	0.333
Bromovaleryl urea (yes)	9	0	0.061
Diprophylline (yes)	5	2	0.904
Pseudoephedrine hydrochloride (yes)	3	3	0.291

## DISCUSSION

The number of patients transported by ambulance owing to overdose of OTC drugs is increasing each year, with some patients experiencing repeated overdoses.^3^ In Japan, the proportion of patients who are primarily dependent on OTC drugs among those with drug‐related disorders visiting psychiatric facilities increased approximately sixfold between 2012 and 2020, and approximately 40% of patients <20 years of age who were treated by psychiatrists for drug‐related disorders had a history of OTC drugs abuse.^3^ In this study, 33 patients (26.6%) also had a history of habitual OTC drugs overdose.

At first glance, it may seem that the patients are not lonely, given that the majority of the patients in our study (87.9%) participated in social activities and most of them (83.1%) lived with their families or partners. However, they attempted self‐harm or suicide, and tried to alleviate their pain and escape from reality by overdosing with OTC drugs (Table 1). It is possible that they may have experienced feelings of isolation within their homes and in wider society due to a lack of sufficient time or opportunity for active conversation, even when housemates or family members were present. As prior research supporting this, the 2021 National High School Student Survey on Drug Abuse and Lifestyle reported that high school students with a history of OTC drugs abuse were characterized by less sleep, less frequent breakfast, less frequent dinner with the whole family, more time spent without adults, few friends to hang out or discuss problems with, not talking to their parents about problems, and a higher rate of prolonged Internet use (≥6 h per day) compared to those without a drug abuse history. In the future, a more detailed survey of the circumstances surrounding patients who have overdosed on OTC drugs may facilitate a deeper understanding of the challenges they face and enable them to communicate these challenges to their families and supporters, thereby enhancing the environment for preventing overdose of OTC drugs.

The dissemination of drug information and impressions of drug use by influential people on social media and other media plays a part in spreading information to individuals in a variety of wider geographical areas.^6^ In this study, the habitual group was significantly more likely to actively seek drug‐related information on the internet and share it with friends. Adolescents often encounter trials and tribulations that may lead to risky behaviors, such as drug use, in search of social connections and solutions. Isolated adolescents who are unable to form positive relationships with their families and others tend to seek self‐identity, peer relationships, acceptance, and approval through social media.^7^ The process of disclosing their own drug use behaviors online to connect with peers under similar circumstances and sharing information to gain acceptance and approval from peers may have contributed to the prevalence of OTC drugs overdoses among many adolescents.

In this study, 87.9% of patients in the habitual group purchased OTC drugs from physical stores. Similarly, the survey conducted by Matsumoto et al.^8^ in 2022 on drug‐related psychiatric disorders in psychiatric facilities nationwide revealed that pharmacies (71.5%) and drugstores (22.2%) were the most common routes for obtaining OTC drugs compared with the Internet (16.4%). According to the results of a nationwide survey conducted by the Ministry of Health, Labor and Welfare in 2022,^9^ the most common reason for purchasing OTC drugs in stores (pharmacies, drugstores, etc.) was “immediate availability and use” (73.7%), followed by “actual selection in person” (64.2%). This suggests that the “immediacy” of the purchase is one of the reasons why people prefer buying from a physical store. However, this also suggests that pharmacists can act as gatekeepers in physical stores and interact with customers, potentially preventing OTC drugs abuse among adolescents. Further detailed research on the route of drug acquisition is crucial for developing preventive measures against OTC drugs overdoses. Conversely, patients in the nonhabitual group were more likely to use drugs that were available nearby, such as in the possession of housemates. Antipyretic analgesics were selected significantly more frequently by the nonhabitual group. Antipyretic analgesics contain ingredients such as caffeine and acetaminophen, which can cause organ damage if overdosed, therefore strict vigilance on medicines, including OTC drugs, by housemates may be an important aspect of preventing overdoses.

Patients in the nonhabitual group overdosed mainly as a means of self‐harm or suicide attempts, whereas those in the habitual group overdosed significantly for reasons other than self‐harm/suicide, including relaxation. Antitussives, expectorants, and antihistamines were selected significantly more often by the habitual group. Antitussives, expectorants, and antihistamines have different effects depending on the dose, and their use for relaxation is a worldwide concern.^10^, ^11^, ^12^, ^13^, ^14^ Both dextromethorphan,^15^, ^16^ the main ingredient in abused antitussives, and diphenhydramine,^17^ an antihistamine, reportedly cause dependence on repeated abuse for recreational purposes. Dextromethorphan‐dependent patients are at a particularly high risk of relapse,^15^ with a reported relapse rate of 89.29% after 1 year. This rate is much higher than that in opiate‐ and alcohol‐dependent patients (>65%) and in cocaine‐ and marijuana‐dependent patients (>75%). Dextromethorphan, an N‐methyl‐D‐aspartate receptor antagonist, belongs to a group of substances with psychoactive (dissociative) effects similar to those of lysergic acid diethylamide and ketamine.^18^ In the etiology of addictive behavior, gamma‐aminobutyric acid/antiglutamatergic mechanisms of action play an important role in the development of dependence. With repeated abuse, the cravings for addictive substances become compulsively repetitive and uncontrollable, leading to withdrawal symptoms with autonomic symptoms and tolerance.^16^ Although it is still unclear how antihistamines act in relation to repeated abusive behavior, it has been reported that increased dopaminergic transmission in the mesolimbic system, which is thought to be involved in the control of emotions, memory, reward systems, attention, and motivation, may be involved.^17^

This study revealed that one of the contributing factors to overdosing on OTC drugs is the psychological isolation experienced by some young people despite their social lives. It is our contention that in order to avoid exacerbating the self‐isolation behavior of these patients, it is imperative that we are attuned to their feelings. In order to do this, it is first necessary for us to ascertain their reasons for using OTC drugs. Then, the support—medical, administrative, educational, or all—that the patient needs must be identified. In addition, if repeated overdose behavior is in part a craving for addictive substances,^16^ then psychotherapy, pharmacotherapy, or treatment should be sought rather than regulation and punishment. Currently, treatment programs for OTC drugs dependence remain lacking, and they must be established with the cooperation of medical, government, and educational institutions.

## LIMITATIONS

There were a few limitations in this study. First, it is important to note that these results are based on a limited sample of only eight facilities, and it is possible that there are many more cases of OTC drugs overdose across the country. Additionally, it is likely that there are cases that have not been reported to medical facilities. In the future, conducting detailed surveys for each of various districts would be desirable. We are unable to identify the same patient who overdosed repeatedly during this period because patients' identity information was not collected during data collection. In addition, it included a group of patients transported to a limited number of facilities. Although poisoning patients should be managed at tertiary emergency centers, some patients were transported to secondary emergency facilities instead. Since many of the collaborating institutions in this study were tertiary facilities, patients of mild severity might have been missed.

## CONCLUSION

To prevent new overdoses of OTC drugs, continued detailed epidemiologic studies of patient backgrounds and drug acquisition routes, and investigation of the components of OTC drugs that cause dependency are necessary. Repeated overdoses may be due to dependency syndrome. The establishment of treatment programs for repeated overdoses is crucial in the future.

## AUTHOR CONTRIBUTIONS

Ryoko Kyan designed the study, developed the main conceptual ideas, and drafted the manuscript with assistance from Yoshito Kamijo, Takuya Shimane, Toshihiko Matsumoto, and Michiko Takai. Ryoko Kyan, Saeko Kohara, Hidetada Fukushima, Shogo Narumi, Takuyo Chiba, Toshiki Sera, Norio Otani, and Yasumasa Iwasaki collected the data. Yoshito Kamijo, Takuya Shimane, Toshihiko Matsumoto, and Michiko Takai assisted in the interpretation of the results.

## CONFLICT OF INTEREST STATEMENT

The authors declare no conflict of interest.

## ETHICS APPROVAL STATEMENT

The research related to human use complied with all the relevant national regulations, institutional policies and in accordance the tenets of the Helsinki Declaration, and has been approved by the Institutional Review Board of the Ethics Committee of the Saitama Medical University Hospital (2021‐064).

## PATIENT CONSENT STATEMENT

All study participants, or their legal guardian, provided informed written consent prior to study enrollment.

## Data Availability

Raw data were generated at Saitama Medical University Hospital. Derived data supporting the findings of this study are available from the corresponding author R.K. on request.
