# Correction to “Prospective multicenter study of the epidemiological features of emergency patients with overdose of over‐the‐counter drugs in Japan”

**DOI:** 10.1002/pcn5.70034

**Published:** 2024-11-07

Kyan R, Kamijo Y, Kohara S, Takai M, Shimane T, Matsumoto T, et al. Prospective multicenter study ofthe epidemiological features of emergency patients withoverdose of over‐the‐counter drugs in Japan. Psychiatry Clin Neurosci Rep. 2024;3:e225. https://doi.org/10.1002/pcn5.225

In the ‘Methods’ section, under the subsection ‘Measures’, the last sentence reads: “This study was approved by the Institutional Review Board of the Ethics Committee of the Saitama Medical University Hospital (2021‐064)”. The ethics review number is incorrect.

This should have read: “This study was approved by the Institutional Review Board of the Ethics Committee of the Saitama Medical University Hospital (Byo2021‐004).”

We apologize for this error.

