# Correction to “Prospective multicenter study of the epidemiological features of emergency patients with overdose of over‐the‐counter drugs in Japan.”

**DOI:** 10.1002/pcn5.70069

**Published:** 2025-02-19

Kyan R, Kamijo Y, Kohara S, Takai M, Shimane T, Matsumoto T, et al. Prospective multicenter study of the epidemiological features of emergency patients with overdose of over‐the‐counter drugs in Japan. Psychiatry Clin Neurosci Rep. 2024; 3:e225. https://doi.org/10.1002/pcn5.225.

In Table 1, the data for ‘Internet’ in the section ‘Routes of access to information (131 in total)’ were inadvertently missed out due to a misalignment in the rows.

**Table 1:** .

		The non‐habitual *n* = 91	The habitual *n* = 33	P‐value
Age	;mean	27.02	21.82	0.037
Sex	;female	70	28	0.338
Occupation	;yes	76	27	0.824
Housemate	;yes	74	29	0.602
Mental problems	;yes	50	21	0.387
Social history				
Alcohol	;yes	50	12	0.083
Smoke	;yes	18	2	0.088
Concomitant drugs/substances	;yes	35	12	0.732
Route of access to drugs (total 129)				
Physical store	;yes	58	29	0.009
Family owned	;yes	26	2	0.008
Internet purchase	;yes	8	4	0.579
Other	;yes	1	1	0.451
Route of access to information (total 131)				
Internet	;yes	30	19	0.013
Physical store	;yes	22	3	0.064
Social Networking System	;yes	13	9	0.094
Friends	;yes	0	6	<0.001
Other	;yes	24	5	0.192
Purpose of use (total 191)				
Suicide/self‐injury	;yes	78	20	0.002
Other	;yes	15	23	<0.001
Escapism	;yes	5	6	
Relief of mental distress	;yes	7	7	
Relaxation	;yes	2	13	
Nothing in particular.	;yes	2	3	

Below is the correct Table 1.

We apologize for this error.

